# Skin‐Inspired Piezoelectric Tactile Sensor Array with Crosstalk‐Free Row+Column Electrodes for Spatiotemporally Distinguishing Diverse Stimuli

**DOI:** 10.1002/advs.202002817

**Published:** 2021-01-06

**Authors:** Weikang Lin, Biao Wang, Guoxiang Peng, Yao Shan, Hong Hu, Zhengbao Yang

**Affiliations:** ^1^ Department of Mechanical Engineering City University of Hong Kong Tat Chee Avenue Kowloon Hong Kong China; ^2^ School of Mechanical Engineering and Automation Harbin Institute of Technology Shenzhen 518055 China; ^3^ Shenzhen Research Institute of City University of Hong Kong Shenzhen 518057 China

**Keywords:** crosstalk‐free, piezoelectric sensor, pressure and bending sensing, real‐time monitoring, tactile sensor array

## Abstract

Real‐time detection and differentiation of diverse external stimuli with one tactile senor remains a huge challenge and largely restricts the development of electronic skins. Although different sensors have been described based on piezoresistivity, capacitance, and triboelectricity, and these devices are promising for tactile systems, there are few, if any, piezoelectric sensors to be able to distinguish diverse stimuli in real time. Here, a human skin‐inspired piezoelectric tactile sensor array constructed with a multilayer structure and row+column electrodes is reported. Integrated with a signal processor and a logical algorithm, the tactile sensor array achieves to sense and distinguish the magnitude, positions, and modes of diverse external stimuli, including gentle slipping, touching, and bending, in real time. Besides, the unique design overcomes the crosstalk issues existing in other sensors. Pressure sensing and bending sensing tests show that the proposed tactile sensor array possesses the characteristics of high sensitivity (7.7 mV kPa^−1^), long‐term durability (80 000 cycles), and rapid response time (10 ms) (less than human skin). The tactile sensor array also shows a superior scalability and ease of massive fabrication. Its ability of real‐time detection and differentiation of diverse stimuli for health monitoring, detection of animal movements, and robots is demonstrated.

## Graphical abstract

Human skin‐inspired piezoelectric tactile sensor array can sense and distinguish the magnitude, positions, and modes of diverse external stimuli in real time. The dual‐layer comb structures of the sensor array with row+column electrodes eliminate crosstalk and reduce the number of connection wires. It excavates enormous applications in various settings, such as health monitoring, detection of animal movements, and robots.

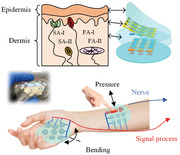

## 1 Introduction

**Figure 1:**
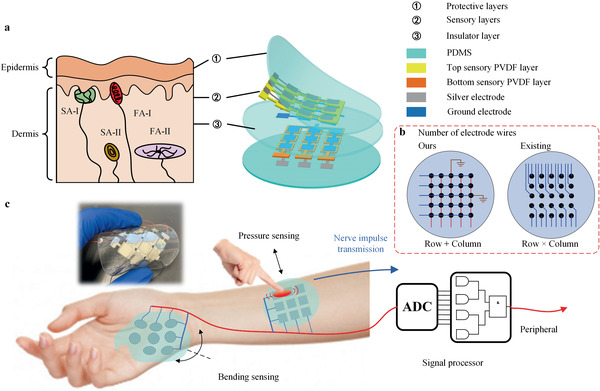
Schematic of the skin‐inspired piezoelectric tactile sensor array. a) Illustration of the human skin structure (left) and the skin‐inspired tactile sensor array (right). b) Wiring connection. For an *n* × *m* sensing array (*n* is the row and *m* is column), only (*m* + *n* + 2) wires are needed in our design. In contrast *n* × *m* + 1 electrode wires are needed in other structures.^[^ ^19^, ^22^, ^23^, ^26^, ^39^ ^]^ c) Schematic of the real‐time tactile sensor array system. The tactile sensor array attached to human skin can detect diverse external stimuli and transfer them to the signal processor and peripheral. After reading the signal, the peripheral integrated with a logical algorithm will recognize the positions, magnitude, and modes of the stimuli in real time.

Human skin is a very amazing sensor that enables simultaneously detecting the intensity and modes of diverse stimuli, including pressing, tapping, slipping, and bending. This ability mainly owes to four mechanoreceptors (SA‐I, II, and FA‐I, II) scattering in different regions of the human skin (**Figure** **1**a).^[^ ^1^, ^2^ ^]^ The mechanoreceptors receive the external stimuli and convert them to electronic signals. The ensemble information from these four receptors is subsequently interpreted by the human brain as body positions, object sizes, shapes, texture, etc.^[^ ^2^, ^3^ ^]^ The disabled without limbs suffer much from the lack of tactile sensing capability. This issue is relieved by the latest development of smart prosthetics integrated electronic skins, which helps the disabled to regain the human tactile system.^[^ ^4^, ^5^ ^]^ The flexible artificial electronic skins integrating with tactile sensors have a huge potential in reconstructing the human tactile system, and enable users to operate more naturally by sensing objects and grip forces.^[^ ^6^, ^7^, ^8^ ^]^

To monitor and recognize diverse mechanical stimuli for electronic skins, research communities have developed various tactile sensors. These sensors resort to different physical transduction mechanisms including resistivity (i.e., contact resistance),^[^ ^9^, ^10^ ^]^ piezoresistivity,^[^ ^11^, ^12^, ^13^ ^]^ capacitance,^[^ ^14^, ^15^, ^16^, ^17^ ^]^ piezoelectricity,^[^ ^18^, ^19^, ^20^, ^21^ ^]^ and triboelectricity.^[^ ^22^, ^23^, ^24^ ^]^ Moreover, efforts have been dedicated to designing microstructures, such as pyramids,^[^ ^17^, ^25^ ^]^ interlocking,^[^ ^9^, ^18^, ^26^ ^]^ and hollow‐sphere^[^ ^11^, ^27^ ^]^ to remarkably improve the sensitivity and broaden detection modes of tactile sensors, including normal force,^[^ ^13^, ^14^, ^15^, ^16^, ^17^, ^18^, ^19^, ^20^, ^21^, ^22^, ^23^, ^28^ ^]^ shear force,^[^ ^9^, ^10^, ^14^ ^]^ bending strain,^[^ ^9^, ^18^, ^28^, ^29^ ^]^ temperature,^[^ ^30^, ^31^ ^]^ and humidity.^[^ ^31^, ^32^ ^]^ Besides, larger‐area,^[^ ^7^ ^]^ high‐resolution,^[^ ^9^ ^]^ and flexibility^[^ ^33^, ^34^ ^]^ are also the desirable characteristics of tactile sensors for artificial electronic skin.

Despite the notable achievements mentioned above, smart prosthetics integrating with electronic skins are not capable of completely replacing human limbs and human skin due to the single function of the tactile sensors and the disability of distinguishing different stimuli in real time. As stated by Prof. Zhenan Bao, “the sensor field has been shown to be uniform across multiple loading conditions, making it difficult to distinguish between them.”^[^ ^6^, ^14^ ^]^ Furthermore, the development of larger‐area and high‐resolution electronic devices has been restricted by the required large number of electrode wires. Even though row+column (i.e., *n* + *m*) structures (Figure 1a) considerably reduce the number of wires in the capacitive sensors,^[^ ^15^ ^]^ this strategy has not been used in piezoelectric sensors because the wiring connection causes crosstalk.^[^ ^35^ ^]^ To eliminate the crosstalk issue, every sensing element needs an individual electrode and wire connection. Consequently, an *n* × *m* sensor array needs *n* × *m* wires, which product number of wires causes many troubles in sensor array fabrication and miniaturization.

Here, we propose a new piezoelectric flexible multifunctional tactile sensor array that is able to sense and differentiate the magnitude, positions, and modes of diverse external stimuli in real time (comparison between other tactile sensors and ours can be referred to Table S1, Supporting Information). We develop a simple but effective electrode topology that is crosstalk‐free and only requires *n* + *m* wires for an *n* × *m* sensor array. Figure 1a illustrates the tactile sensor design inspired by human skin. Anatomy tells that our human skin is a multilayer structure and each layer has its unique function.^[^ ^36^ ^]^ Specifically, the epidermis forms a protective layer; the dermis serves as cushioning the body from stress and strain and sensing touch and heat. The epidermis and dermis are slightly connected by a layer called the basement membrane. Mimicking human skins, we design the tactile sensor array with two protective layers, two sensory layers, and one insulative layer (Figure 1a). We expect to sense gentle slip stimuli, touch stimuli, and bending stimuli with high sensitivity and rapid response time. The dual‐layer comb structures of the sensory layers with row+column electrodes eliminate crosstalk and reduce the number of connection wires. The simple electrode wire topology has two advantages: 1) A large number of sensor pixels can be fabricated with a small quantity of wires; 2) individual sensor array can be assembled together with no need of rewiring. We demonstrate the skin‐inspired tactile sensor array for health monitoring, detection of animal movements, and robots.

## 2 Results and Discussion

### 2.1 Design of Human Skin‐Inspired Tactile Sensor Array

Inspired by the multilayer structure of human skin, we design the tactile sensor array with five layers, namely, two protective layers, two sensory layers, and one insulative layer (Figure 1a). The protective layers made of polydimethylsiloxane (PDMS) (*E* = 2.6 MPa, thickness 100 µm) mimics the epidermis of human skin to transmit external stimuli and protect the inner structure. The polyvinylidene fluoride (PVDF)‐made sensory layers (*E* = 3 GPa, thickness 28 µm), which lie at different depths in the sensor array, are used to receive the stimuli from the protective layers and convert them into electrical signals. This process acts similarly as mechanoreceptors (FA‐I, II and SA‐I, II) in human skin. Analogous to human skin, we name a two‐layer structure, as the top PVDF sensory and the bottom PVDF sensory layer in the tactile sensor to represent their positions in the structure. The PDMS insulative layer (thickness 500 µm) serves in two aspects. First, it avoids the crosstalk of two PVDF layers; second, the thick middle layer guarantees the stress neutral layer of the device far away from both PVDF layers, thus enabling each PVDF layer to be only tensile or compressive under bending stimuli and enhancing the output voltage. PDMS is selected since it is a toxic‐free, biocompatible, and low‐cost material^[^ ^37^ ^]^ and it mimics the skin tissues and dermis. PVDF has advantages such as ultratransparency, high flexibility, and lead‐free biocompatibility.^[^ ^38^ ^]^

In addition to the bionic multilayer structure in our design, the layout of the PVDF layers is the key to recognize diverse stimuli modes, avoid crosstalk interferences, and reduce the number of wires. In Figure 1a, we demonstrate a 3 × 3 tactile sensor array. Each sensory layer is designed into a comb pattern. For each sensory PVDF layer, there are two electrodes attached to both sides, respectively. The electrode of one side includes three separate parts and acts as three detection channels, while that on the other side is made united and acts as a ground electrode. Each channel connects to one wire. Thus, for each channel, it avoids the crosstalk since other channels do not share the same electrodes with it. The channels of the top sensory and bottom sensory layers are arranged to be perpendicular (i.e., row+column structure). In the following, we will show this arrangement successfully achieves the differentiation of diverse external stimuli. The comb design, on the one hand, avoids the local deformation of the three channels and transfers the stress more uniformly. On the other hand, it allows the electrodes for the channels on one side to connect with each other and reduce the number of wires. As illustrated in Figure 1b, a 5 × 5 array, for example, for *n* × *m* sensing units (*n* is the row and m is column), only (*m* + *n* + 2) wires are needed in our design. In contrast, *n* × *m* + 1 electrode wires are needed in other structures.^[^ ^19^, ^22^, ^23^, ^26^, ^39^ ^]^ Another characteristic of the proposed design is its self‐powered ability due to the piezoelectric transduction mechanism that receives much interest in the large‐area and high‐resolution electronic devices.^[^ ^7^ ^]^

Furthermore, we develop a real‐time tactile system based on the tactile sensor array. As shown in Figure 1c, this system mimics the biological response process of the human being.^[^ ^12^ ^]^ First, the PVDF films in the sensor array receive the external stimuli from the protective layers. Then, the stimuli will be converted to the electrical signal via the direct piezoelectric effect. Third, the signal is transferred to the peripheral, such as a computer, after signal processing. By reading the signal, the peripheral with the configuration of a logical algorithm will recognize the positions, magnitude, and modes of the stimuli in real time.

### 2.2 Working Mechanism

**Figure 2:**
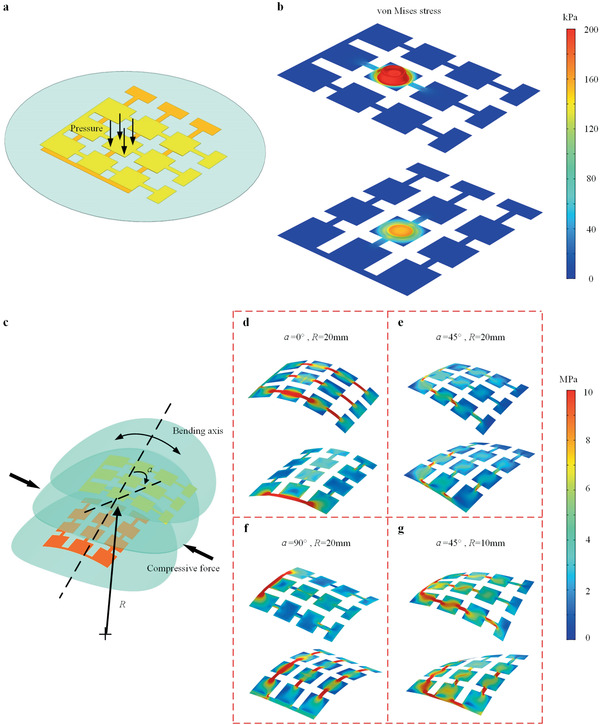
Working mechanism of the tactile sensor array under different stimuli modes. The finite element simulation describes the stress distribution of the sensor array under different types of external stimuli. a) Schematic of the tactile sensor array under pressure stimulus mode. b) The stress analysis shows that the signal mainly occurs at the pressure zone and the signal of the top PVDF sensory layer is larger than that of the bottom PVDF sensory layer. c) Schematic of the tactile sensor array under bending stimulus mode. d–g) The stress of the surface layer decrease with a larger bending angle, while the case of the deep layer shows the opposite trend. The stress of both layers is close to each other at 45° of bending angle. Besides, a larger bending radius leads to larger stress.

**Figure 3:**
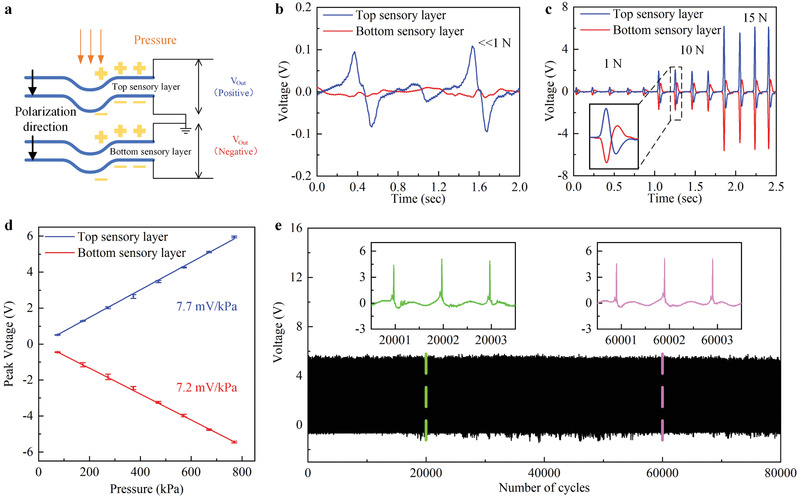
Experimental results of the tactile sensor array under the pressure stimuli mode. The measured signal is generated by the middle sensing unit. a) The direction of the polarization of the piezoelectric sensory films and the circuit diagram of the tactile sensor array. b) The real‐time waveform of the output voltage under gentle slip stimuli. c) The real‐time waveform of the output voltage under different normal force amplitudes with a frequency of 5 Hz. d) Output peak voltage with error bar versus normal force with the amplitude from 80 to 750 kPa. The sensor array shows a high linearity, low error, and high sensitivity. e) The durability test. The top PVDF sensory responses stably after 80 000 cycles under a normal force of an amplitude of 15 N and frequency of 30 Hz.

To theoretically analyze the working mechanism of the tactile sensor array, we conduct finite element (FE) simulations. The sensor array undergoes two stimuli modes under different types of loads. In the first stimuli mode, as shown in **Figure** **2**a, the pressure is applied to a sensing pixel. Here we select the middle pixel as a representative. Both layers are thus in the compressive stress state. Figure 2b shows the stress distribution of the top sensory and bottom sensory PVDF layers. It can be observed that the maximum stress occurs at the pressure‐bearing pixel in each layer. The stress of the top sensory layer is higher than that of the bottom sensory layer. With the consideration of the stress state, the piezoelectric constitutive relations, the direction of the polarization, and circuit connection (**Figure** **3**a), we can predict that the output voltage of the two layers keeps opposite and the signal of the top sensory layer is higher. In the bending stimuli mode, as we mentioned above, the neutral layer lies in the PDMS insulative substrate, thus leading to the opposite stress state for the PVDF layers. Besides, under bending deformation, the output voltage generated by the PVDF layers is simultaneously determined by the bending direction and bending radius. Figure 2c defines the angle of the bending direction *α* and bending radius *R*. From Figure 2d–g, we interpret that the stress of the top sensory layer decreases with a large bending angle, while the bottom sensory layer shows an opposite trend. The stress of both layers is close to each other at 45° bending angle. The reason is that the top sensory layer bends effectively at 0°, while the most effective bending direction is 90° for the bottom sensory layer. At 45°, the structure and its applied loads are approximately symmetric, thus the stress distribution of both layers is close. As expected, a larger bending radius leads to smaller stress. Considering the stress state, the piezoelectric constitutive relations, the direction of the polarization, and circuit connection (Figure 3a), the output voltage of the two layers in the bending stimuli mode will keep pace with each other and the voltage signal depends on both the bending angle and bending radius. We also investigate the effect of the thickness and Young's modulus of the protective and insulative layers on the stress field. Figure S1 (Supporting Information) shows the stress distribution of both top and bottom sensory layers when the thickness of two protective layers changes with the unchanged insulative layer. Under the same bending direction and bending radius, the stress with thicker protective layers is slightly higher. The stress increase comes from the larger stiffness of the device owing to the thicker protective layers. The bigger Young's modulus of protective and insulative layers will also cause the stiffer structure. Thus, the stress increases in the top and bottom sensory layers with stiffer protective layers, as illustrated in Figure S2 (Supporting Information). With the consideration of the direct piezoelectric effect, we can infer that the output voltage in these two sensory layers shows the same trend when the thickness and Young's modulus of the protective and insulative layers change.

### 2.3 Pressure Sensing

Our skin‐inspired tactile sensor array can measure both the magnitude and positions of pressure stimuli. Figure 3 shows the direction of polarization and circuit diagram of the sensor array, and the electrical responses caused by pressure stimuli. The bottom silver electrode of the top sensory layer and the top silver electrode of the bottom sensory layer are grounded. As mentioned in Section 2.1, the top electrode of the top sensory layer and the bottom electrode of the bottom sensory, respectively, contain three separate channels. When the pressure is applied to a pixel, one of the channels in both the top sensory layer and the bottom sensory layer will respond. The cross of two channels in the two layers represents the position applied to pressure stimulus. Excited by gentle slip stimuli (Figure 3b), responses of both PVDF layers at this pixel are small, but the output voltage of the top PVDF sensory layer is much larger than that of the bottom sensory layer (0.1 V vs 0.02 V), attributed to the thick middle insulative PDMS layer that absorbs much energy. In this experiment, a steel bar used to stimulate the surface of the tactile sensor array is connected to the ground electrode, which eliminates the triboelectric effect on piezoelectric signal output.^[^ ^40^ ^]^ For touch stimuli, we test the sensor with different pressure amplitudes. As shown in Figure 3b, a large pressure corresponds to the large output voltage and the real‐time waveform of the output voltage clearly shows the opposite phase of the two layers. This phenomenon is expected and has been explained previously in the FE simulations. Besides, it can be observed, again, that the signal of the top PVDF sensory layer is larger due to the existence of the insulative layer. Figure 3c shows the waveform of the output voltage under different normal force amplitudes with a frequency of 5 Hz. Figure 3d presents the relations of the peak output voltage to different amplitudes of pressure with a frequency of 5 Hz for the top and bottom sensory PVDF layers. The sensor array shows high linearity, low error and high sensitivity (7.7 and 7.2 mV kPa^−1^ for the top sensory layer and the bottom sensory layer, respectively). The difference in the sensitivity for both sensory layers is attributed to the insulative layer since the top sensory layer absorbs more deformation from external stimuli. The response time of the sensor array is fast (Figure S3, Supporting Information). Excited by a load of 15 N and 5 Hz, the response time is 10 ms, which has surpassed that of the human skin (about 15 ms).^[^ ^6^ ^]^ Our sensor array also shows long‐term durability and stability. It outputs stable responses after 80 000 cycles under 15 N force (Figure 3e). After 80000 cycles, the output voltage begins to gradually decrease (Figure S4a, Supporting Information). The SEM images of the silver electrode surface before and after long‐term durability test are shown in Figure S4b–d (Supporting Information). Before the durability test, the surface of the silver electrode is flat and unit (Figure S4b, Supporting Information). Since the top sensory layer is close to the external stimuli and the protective PDMS layer is thin, the silver electrode surface of the top sensory layer suffers damage after the 120000 cycles (Figure S4c, Supporting Information), while the that of the bottom sensory layer is still intact due to the thick insulative PDMS layer (Figure S4d, Supporting Information).

### 2.4 Bending Sensing

**Figure 4:**
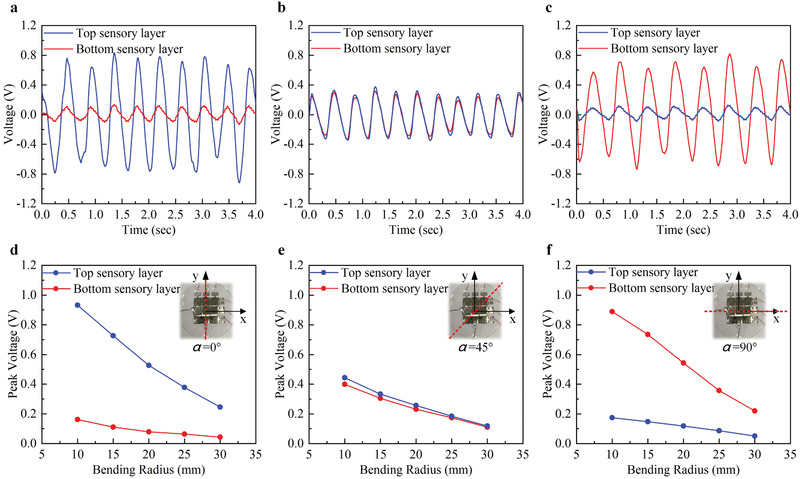
Experimental results of the tactile sensor array under the bending stimuli mode. a–c) The real‐time waveform of the output voltage bending at 0°, 45°, and 90° with the bending radius of 15 mm. d–f) The output peak voltage versus bending radius.

The task of bending sensing is to simultaneously recognize the bending direction *α* and bending radius *R* (Figure 2c). Here, we first experimentally obtain the electrical signal under different bending conditions and subsequently establish their relations. **Figure** **4**a–c shows the measured output voltage waveforms of the sensor array bent at 0°, 45°, and 90° with a bending radius of 15 mm. It indicates that a large bending angle lowers the output voltage of the top sensory layer but raise that of the bottom sensory layer, and the output voltage waveforms of the top sensory layer and bottom sensory layer show the same phase. The measured result is similar to the analysis in FE simulations. The trend in Figure 4d–f represents that larger output voltage occurs at a smaller bending radius. Another observation is that the top sensory layer has the highest sensitivity at 0° bending direction and 90° for the bottom sensory layer.

**Figure 5:**
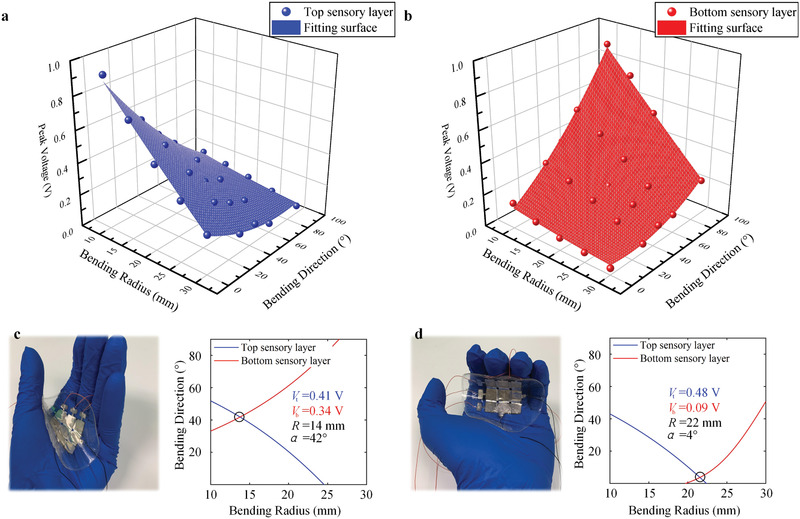
Sensing the bending direction and bending radius. It shows that the tactile sensor array is capable of simultaneously sensing the bending direction and bending radius. a,b) 3D fitting results based on experimental data. c,d) Demonstration of the tactile sensor array to detect the hand deformation via contour lines. Based on the output voltage of the top sensory and bottom sensory layers, two contours are plotted in one plane. The bending direction and bending radius are indicated by the intersection of the contours.

To infer the bending direction *α* and bending radius *R*, we further build the relations of the output voltage to bending direction and bending radius using the experimental results (Tables S2 and S3, Supporting Information). The fitting function is shown in Table S4 (Supporting Information) and the fitting result of the top sensory layer and bottom sensory layer are shown in **Figure** **5**a,b. As shown in Figure 5c,d, the sensor array is held by the human palm. In the first demonstration (Figure 5c), the thumb of a tester moves and we get two voltage contours of the top sensory layer (*V*_t_) and the bottom sensory (*V*_b_). The contours have an intersection, which records the bending direction and bending radius. In this case of Figure 5c, they are 42° and 14 mm, respectively. In the second demo of Figure 5d, the tester moves his other four fingers. We interpret the bending direction and bending radius as 4° and 22 mm.

### 2.5 Multipixel Detection and Large‐Area Scalable Design

**Figure 6:**
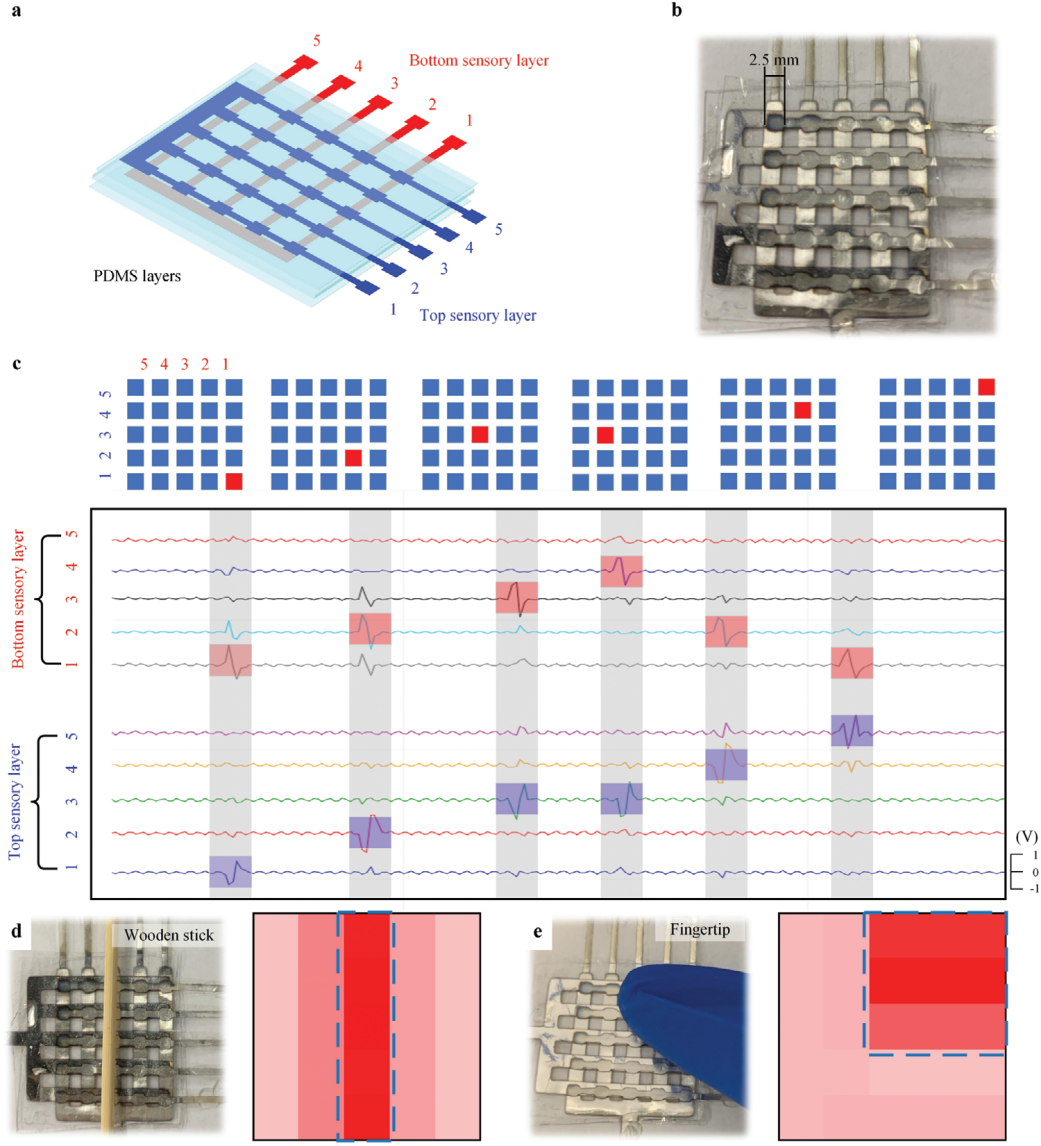
Illustration of a large‐area scalable tactile sensor array and its ability for multipixel detection. The tactile sensor array is suitable for large‐area scalable fabrication and bulk production. a) Schematic of the 5 × 5 bionic tactile sensor array. b) Photograph of the fabricated 5 × 5 tactile sensor array with each unit of 2.5 × 2.5 mm^2^. c) Illustration of pressure stimuli and the corresponding real‐time signal from the sensor array. The sensor array accurately captures the pressure position. d,e) Detection of multiple touchpoints. The profiles of planar pressure intensity show that the sensor array accurately captures multitouch excitations.

To further improve the resolution of a tactile sensor array, a large‐area design is desirable but challenging for existing sensors^[^ ^19^, ^22^, ^23^, ^39^ ^]^ because the increase in sensing pixels usually requires a large number of wires. In contrast, our design can be easily extended to the large‐area sensor array. It does not need numerous wires as well as rewiring. To exemplify this property, a 5 × 5 sensor array is fabricated, as shown in **Figure** **6**a,b. The number of wires used in this sensor array is 12 (compared to 25 for 5 × 5 sensor array). Pressure sensing tests are performed. As illustrated in Figure 6c,d, the location of the pressure stimuli can be easily judged in the cross of the maximum voltage signals of the top sensory layer and bottom sensory layer.

In addition to the single‐pixel stimuli illustrated in pressure and bending sensing experiments, the tactile sensor array also can identify multipixel stimuli. The working mechanism of multipixel pressure sensing is introduced as follows. First, the logical algorithm scans every channel and decides whether the channel is activated by the signal of the top sensory and bottom sensory layers. Considering the fabrication, the threshold is set to 0.4 V. The signal higher than the threshold is considered as activation. If both the top sensory and bottom sensory are activated, the voltage of this intersection is calculated by *V* = (*V*_t_ + *V*_b_)/2, otherwise, the voltage is represented by *V* = min (*V*_t_,*V*_b_), where *V* represents the external stimuli, *V*_t_ and *V*_b_ are the output voltage of the top sensory and bottom sensory PVDF layers, respectively.

Figure 6d,e shows two examples of multitouch stimuli. In Figure 6d, a wooden stick presses the third column of the sensor array. In Figure 6e, the pressure to the top right corner of the sensor array comes from a fingertip. We can observe the magnitude and the locations of the stimuli. The real‐time waveforms of the two cases are shown in Figure S5 (Supporting Information), from which we can see the crosstalk‐free properties of the tactile sensor array.

### 2.6 Real‐Time Differentiation of Diverse External Stimuli

Compared to the existing sensors in the literature, the developed tactile system can detect and differentiate the magnitude, locations, and modes of external stimuli in real time. A comprehensive comparison is tabulated in Table S1 (Supporting Information). The characteristics of our skin‐inspired tactile sensor are summarized as follows: i) Under the gentle slip stimuli mode, the output voltage of the bottom sensory layer is close to zero; ii) under the touch stimuli mode, the output voltage waveforms for the top sensory and bottom sensory layers show 180° phase shift; iii) under the bending mode, the output voltage waveforms for the top sensory and bottom sensory layers are in the same phase.

To achieve real‐time differentiation, a logical circuit is developed in the software LabVIEW 2017. Figure S6 (Supporting Information) shows the logical flow diagram of the algorithm. All pixels are scanned by the logical algorithm. The diagram of the logical circuit based on the algorithm is shown in Figure S7 (Supporting Information). For each pixel, first, the logical circuit simultaneously decides the locations and modes of the external stimuli. When the electrical signal of the top sensory layer is higher than a threshold, which is set to 0.05 V in this study, the channel is considered in an activated state. Then, the circuit will determine whether the stimuli is a gentle slip by reading the data of the bottom sensory layer. If this signal is less than another threshold (i.e., 0.02 V), the logical circuit will consider the stimuli as a gentle slip. Otherwise, the circuit will continue to compare the sign of the signal generated by two layers. The same sign means touch stimuli mode, while the opposite sign corresponds to bending stimuli mode. After the type of external stimuli is identified, we can obtain the pressure value of every pixel using the results in Figure 3d or the bending direction and bending radius via the contours of the fitting polynomial in Figure 5. A real‐time differentiation of gentle slip, touch, and bending stimuli is shown in Video S1 (Supporting Information).

### 2.7 Applications

**Figure 7:**
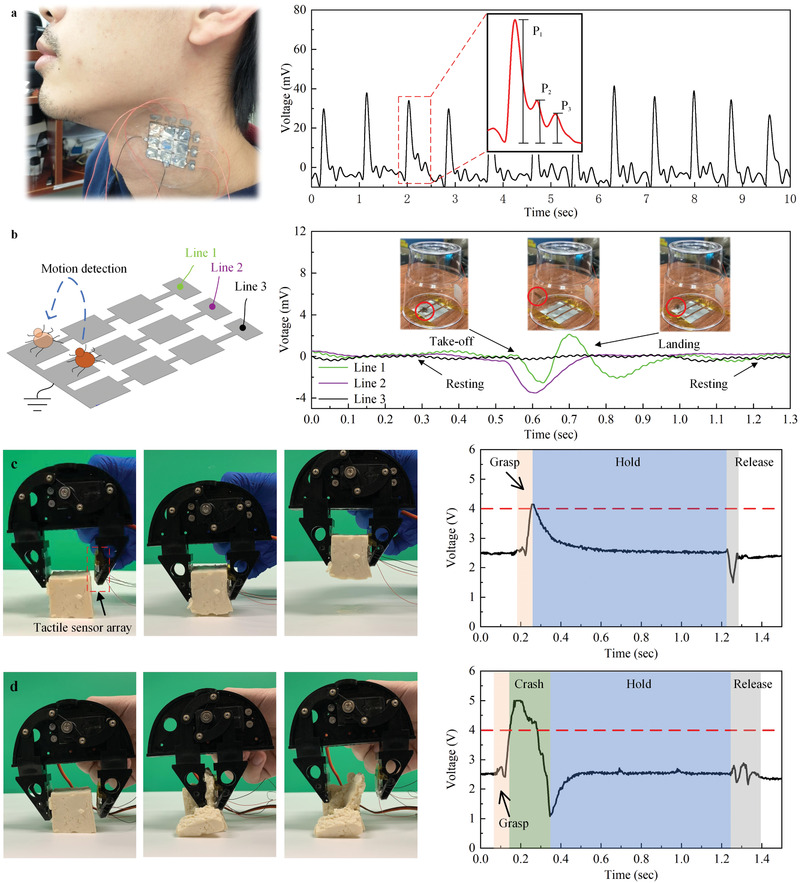
Applications of the tactile sensor array. a) Real‐time detection of the neck arterial pulse. The peaks P_1_, P_2_, and P_3_ are, respectively, the incident, tidal, and diastolic wave. b) Real‐time detection of animal motions. A spider that weighs about 5 mg is used. The voltage signal indicates the take‐off and landing positions of the spider, resting time, and duration of passage, and shows the high sensitivity of the sensor array. c) Demonstration of grasping objects using a robotic hand with a feedback module. The tactile sensor is mounted on the robotic hand and used as real‐time feedback. A piece of soft and fragile tofu keeps integrity during the movement of the robotic hand. The sensor array detects the whole of the grasping process. d) Demonstration of grasping objects using a robotic hand without the proposed sensor array. A piece of soft and fragile tofu damaged during the movement of the robotic hand.

The tactile sensor array has various applications from health monitoring, detection of animal movements to robots. In **Figure** **7**a, the sensor array is used to monitor the subtle physiological signals for a healthy purpose. The sensor array is attached to the bare skin of the neck and accurately detects the weak artery pulse, of which three responses the incident wave P_1_, the tidal wave P_2_, and the diastolic wave P_3_ are recorded.^[^ ^41^ ^]^

The sensor array can be used to detect the movement of small animals. For demonstration, we use a 5 mg weight spider to show the high sensitivity of our device (Figure 7b and Video S2, Supporting Information). To enhance the sensitivity to measure the extremely small pressure from the tiny spider movement, we remove all layers of the tactile sensor except the top sensory layer. Initially, the spider keeps still in the middle of the Line 1 and Line 2. It then takes off toward the wall of a plastic cup and lands on Line 1 to rest. The sensor array accurately captures all behaviors of the spider, including landing positions, resting time, and the duration of the passage. Besides, the signal of the three channels shows no interference with each other. One should note, here, that changing the thickness of the protective and insulative layers is an effective strategy to achieve the adjustment of the sensitivity for sensors. This method is also used in nature. For example, the difference in thickness between human fingertips and palms satisfies diverse sensing. The optimization of the thickness enables the sensor array to broaden the application scenarios.

Finally, we perform a robotic hand experiment as an industrial application. In Figure 7c, the tactile sensor array is mounted on the robotic hand. With the aid of the sensor feedback, the robotic hand correctly interrupts the grasping process and keeps holding when the feedback signal exceeds the 4 V default threshold followed by releasing (Video S3, Supporting Information). A comparative trial without the sensor array as feedback is conducted in Figure 7d. The soft and fragile tofu is damaged without the tactile sensor (Video S4, Supporting Information).

## 3 Conclusion

Sensing and distinguishing diverse external stimuli within one sensor reduces fabrication complexity and alleviates space constraints for electronic skins. The developed skin‐inspired piezoelectric tactile sensor array has the spatiotemporal detection and distinction ability of the magnitude, positions, and modes of diverse external stimuli, including gentle slipping, touching, and bending.

The tactile sensor array is designed to be a multilayer architecture inspired by the structure of human skin. Specifically, two PDMS films serve as the protective layers, two PVDF films as the sensory layers, and one PDMS film as the insulative layer. The working mechanism is theoretically investigated via finite element simulation and verified by experiments. The pressure sensing experiment proves its characteristics of zero crosstalk, rapid response time, high sensitivity, and long‐term durability. The response time is 10 ms, much less than that of the human skin 15 ms. The sensitivity of the top sensory and bottom sensory layer is, respectively, 7.7 and 7.2 mV kPa^−1^. The sensitivity can be tuned via changing the thickness of the protective and insulative layers. The bending sensing experiment shows that we can calculate the bending direction and bending radius using the tactile sensor array. Using the row+column electrodes, this design can be extended to more pixels using a small quantity of wires (*m* + *n* + 2 for *n* × *m* sensing pixels), which enables large‐area scalable fabrication and improves the resolution. The real‐time detection and distinction abilities are demonstrated by monitoring the motions of the neck arterial pulse, the movements of a tiny spider, and the grasping of the robotic hand.

In summary, this study reports a skin‐inspired piezoelectric tactile sensor array with real‐time differentiation ability of diverse external stimuli. Our design eases the challenging tasks in spatiotemporally distinguishing diverse stimuli within one sensor. This research provides a new strategy for tactile sensor design and would broadly benefit many fields, especially for electronic skins, health monitoring, animal movement detection, and robotics.

## 4 Experimental Section

##### Fabrication of PVDF and PDMS Layers

The polarized PVDF films were purchased from Kureha (Kureha Inc., Japan) and PDMS films from Bald Advanced Mat (Hangzhou Bald Advanced Materials Technology Co., China). Silver electrode was first deposited onto the PVDF layers (50 × 50 mm^2^). A comb‐shaped mask made of stainless steel covers one side of the PVDF film to form a specific pattern shown in Figure 1. A target sputtering system (Quorum Technologies Inc., Canada, Quorum Q150TS Dual) was used to deposit a 100 nm silver layer. The procedure of depositing a silver electrode onto the other side is similar, but the mask was removed this time. Then, each PVDF layer was cut into the same pattern as the electrode and connected with wires using silver paste. For the PDMS layers, they were treated with air plasma (HARRICK PLASMA, PDC‐32G) to form a hydrophilic surface, which is a flexible adhesive layer.^[^ ^42^ ^]^ The PDMS layers finally covered the top sensory and bottom sensory PVDF layers like the sandwich. Although the bubbles are observed at the interface of the PVDF layer and PDMS layer (Figure S8a, Supporting Information), the adhesion between these layers is very tight. Therefore, the prototype made by this method works well in the tests. Besides, to eliminate these bubbles, the PDMS solution instead of PDMS films can be used when spin‐coating, as shown in Figure S8b (Supporting Information).

Since the sputtering system is hard to deposit a large‐area silver electrode, the screen printing technology can be used to illustrate its scalability of the design. The method is cost‐effective and suitable for large‐scale manufacturing.^[^ ^43^ ^]^ As an example, an 8 × 8 sensor array was fabricated. In the process, the silver paste was used as the electrode material. Similar to the sputtering process, the comb‐shaped mask with a specific pattern is to form the electrode of one side with eight separate channels using a screen printer (Bojing Printing Equipment Co., China, BJ‐3050). The other silver electrode of the other side was processed similarly, but the mask is different this time. To ensure that the electrodes of both sides of the PVDF are aligned, as shown in Figure S9 (Supporting Information), mark points were added as reference points and the microscope was used to align these points. Since the PDMS is transparent, the same method was also used to keep the top and bottom sensory layers aligned. Then, the PVDF film with electrodes was put in a 60 °C oven for 1 h. This PVDF film was cut into a comb by a Cutting Plotter (GRAPHTEC, Japan, CE6000‐60) and sealed by PMDS films (Figure S10a, Supporting Information). Figure S10b–d (Supporting Information) tests the 8 × 8 tactile sensor array. It shows similar performance as the 5 × 5 tactile sensor array. It is expected that this method can be used to fabricate the tactile sensor array with more and/or small‐size pixels, thus achieving a good scalability and high resolution.

##### Pressure Sensing and Bending Sensing Experimental Setup

Figure S11 (Supporting Information) illustrates the experimental setup of pressure sensing tests. The sensor array was mounted to a high‐precision three‐axis stabilized platform. To ensure the horizontality of the platform, the platform was fixed onto an optical table. A shaker (Gelsonlab, HSPW‐003) driven by a signal generator (RIGOL Technologies, DG1062Z) and power amplifier (Shenzhen TZT Technology Co., Ltd, FPA101A) provides the pressure. The pressure was measured by a high‐resolution strain gauge (SIMBATOUCH, SBT291) which is attached to the bottom of the sensor array. The electrical signal generated by the sensor array was recorded by an oscilloscope (Rohde&Schwarz, RTE 1024).

Figure S12 (Supporting Information) illustrates the experimental setup of bending sensing tests. As shown in Figure S12a (Supporting Information), the circuit connection keeps the same as that in the pressure sensing tests. To generate bending strain in the tactile sensor array, the tactile sensor array was fixed using two fixtures. In the tests, one fixture keeps still, while the other is fixed on a linear motor with 2.5 Hz working frequency. The bending radius of the sensor array was tuned by changing the distance between two fixtures. The electrical signal generated by the sensor array was recorded by an oscilloscope (Rohde&Schwarz, RTE 1024).

All volunteers have known all details about the experiment which is attaching a sensor to the volunteer's neck surface to detect the artery pulses. The experiment results will be used to conduct further research. The Smart Transducers and Vibration Laboratory will ensure the health and safety of all volunteers in the experiment.

All volunteers have agreed to participate in the experiment.

## Conflict of Interest

The authors declare no conflict of interest.

## Supporting information

Supporting Information

Supplemental Video 1

Supplemental Video 2

Supplemental Video 3

Supplemental Video 4
