# Supplementary material for: Skin‐Inspired Piezoelectric Tactile Sensor Array with Crosstalk‐Free Row+Column Electrodes for Spatiotemporally Distinguishing Diverse Stimuli
Source: Adv Sci (Weinh). 2021 Jan 6;8(3):2002817. doi: 10.1002/advs.202002817 (PMC7856889; doi:10.1002/advs.202002817)
Supplement: Supplementary file 1 — Supporting Information [file ADVS-8-2002817-s001.pdf]

WILEY-VCH

© 2020 Wiley-VCH GmbH

ADVANCED SCIENCE
Open Access

Supporting Information

for *Adv. Sci.*, DOI: 10.1002/advs.202002817

Skin-inspired Piezoelectric Tactile Sensor Array with Crosstalk-free Row+column Electrodes for Spatiotemporally Distinguishing Diverse Stimuli

Weikang Lin[#], Biao Wang[#], Guoxiang Peng, Yao Shan, Hong Hu,and Zhengbao Yang**

Supporting Information

Title: Skin-inspired Piezoelectric Tactile Sensor Array with Crosstalk-free Row+column Electrodes for Spatiotemporally Distinguishing Diverse Stimuli

Weikang Lin#, Biao Wang#, Guoxiang Peng, Yao Shan, Hong Hu, Zhengbao Yang**

Contents

Figure S1. The stress distribution with various thickness of the PDMS layers.

Figure S2. The stress distribution with different Young's Modulus of PDMS layers.

Figure S3. The instant response of the tactile sensor array.

Figure S4. The SEM images of the electrode surface before and after long-term durability test.

Figure S5. The real-time output voltage waveform under multi-touch stimuli mode.

Figure S6. Logical flow diagram of a logical logarithm.

Figure S7. Diagram of the logical circuit developed in LabVIEW 2017.

Figure S8. The influence of different packaging methods on the adhesion.

Figure S9. Illustration of the scalability of the design.

Figure S10. The experimental setup for the pressure sensing tests.

Figure S11. The experimental setup for the bending sensing tests.

Table S1. Comparison of the existing tactile sensors with our work.

Table S2. The measured output peak voltage of the top PVDF sensory layer.

Table S3. The measured output peak voltage of the bottom PVDF sensory layer.

Table S4. Fitting equations and the corresponding coefficients.

Video S1. Real-time differentiation of gentle slip, touch and bending stimuli. (speed - 2x)

Video S2. Real-time movement detection of a 5 mg weight spider. (speed - 0.1x)

Video S3. Demonstration of grasping soft and fragile Tofu using a robotic hand with a sensor as a feedback module. (speed - 1x)

Video S4. Demonstration of grasping soft and fragile Tofu using a robotic hand without a feedback module. (speed - 1x)

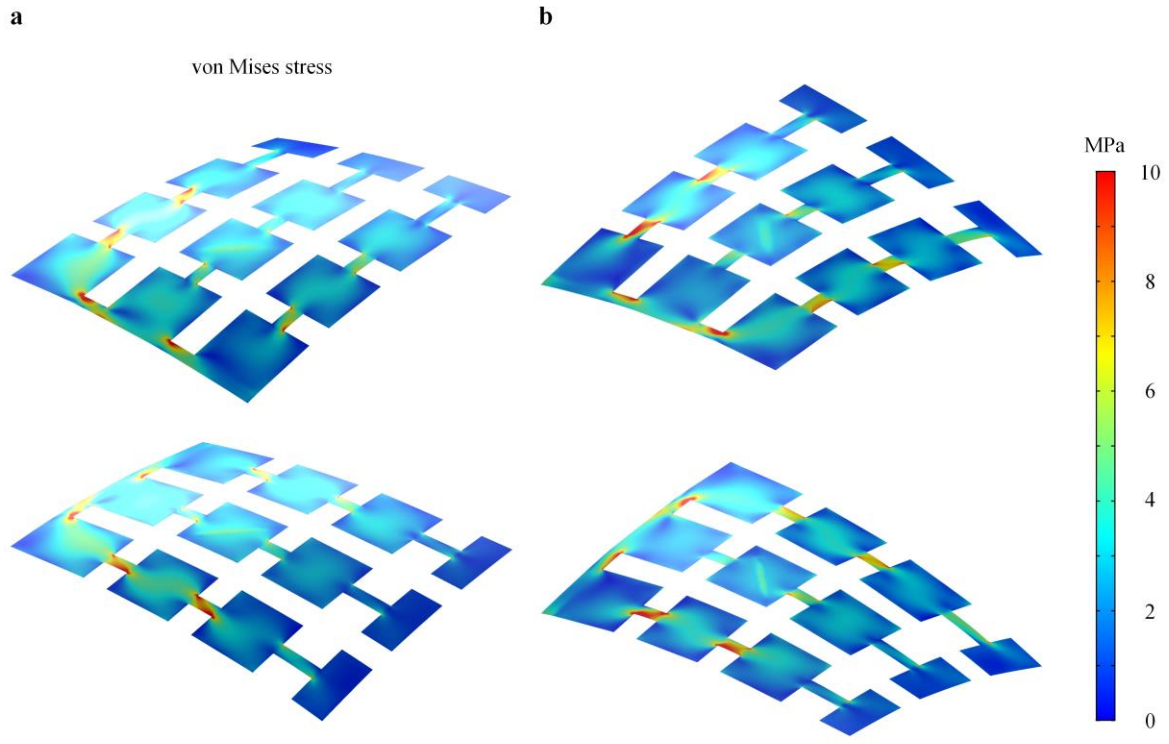

Figure S1. The stress distribution with various thickness of the PDMS layers. Thickness of two protective layers is a) 50 μm; b) 200 μm. The bending direction is 45° and the bending radius is 20 mm.

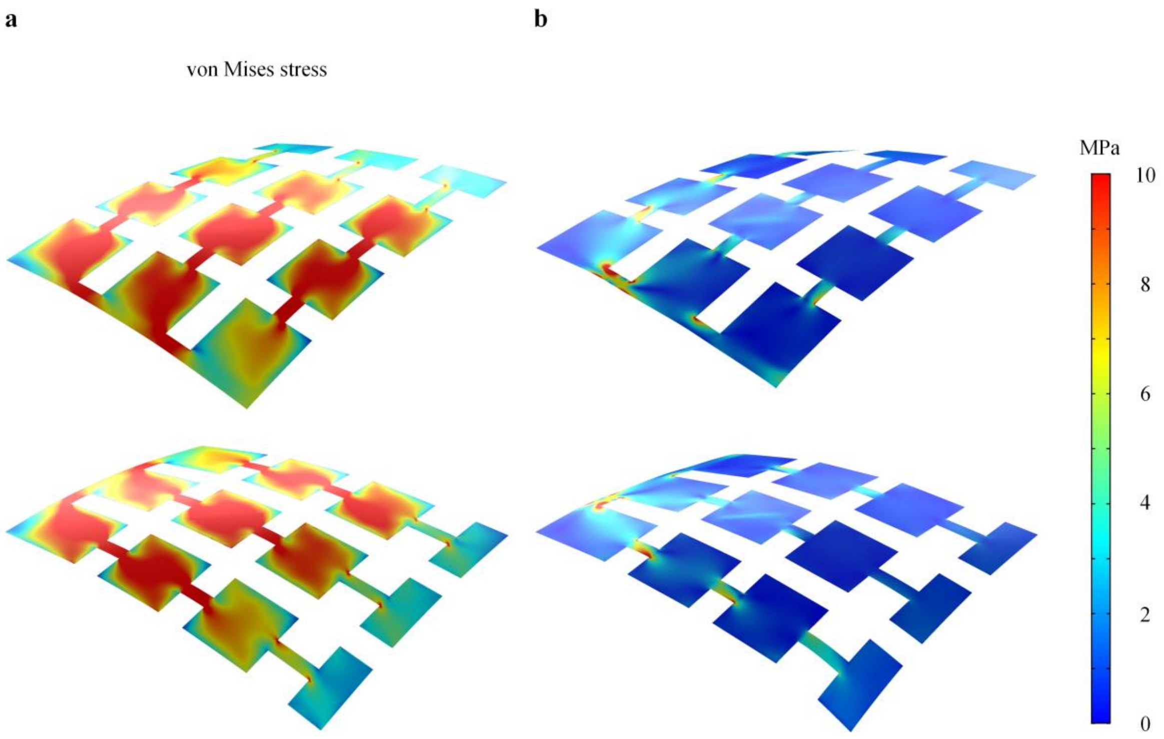

Figure S2. The stress distribution with different Young's Modulus of PDMS layers. Young's Modulus of protective and insulative layers is a) 0.26 MPa; b) 26 MPa. The bending direction is 45° and the bending radius is 20 mm.

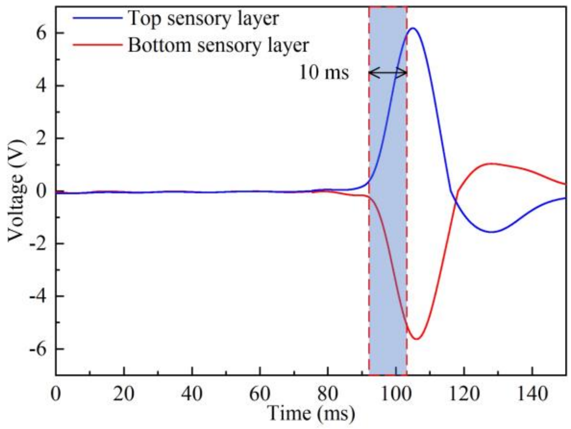

Figure S3. The instant response of the tactile sensor array. The response time is 10 ms, much less than that of the human skin (about 15 ms).

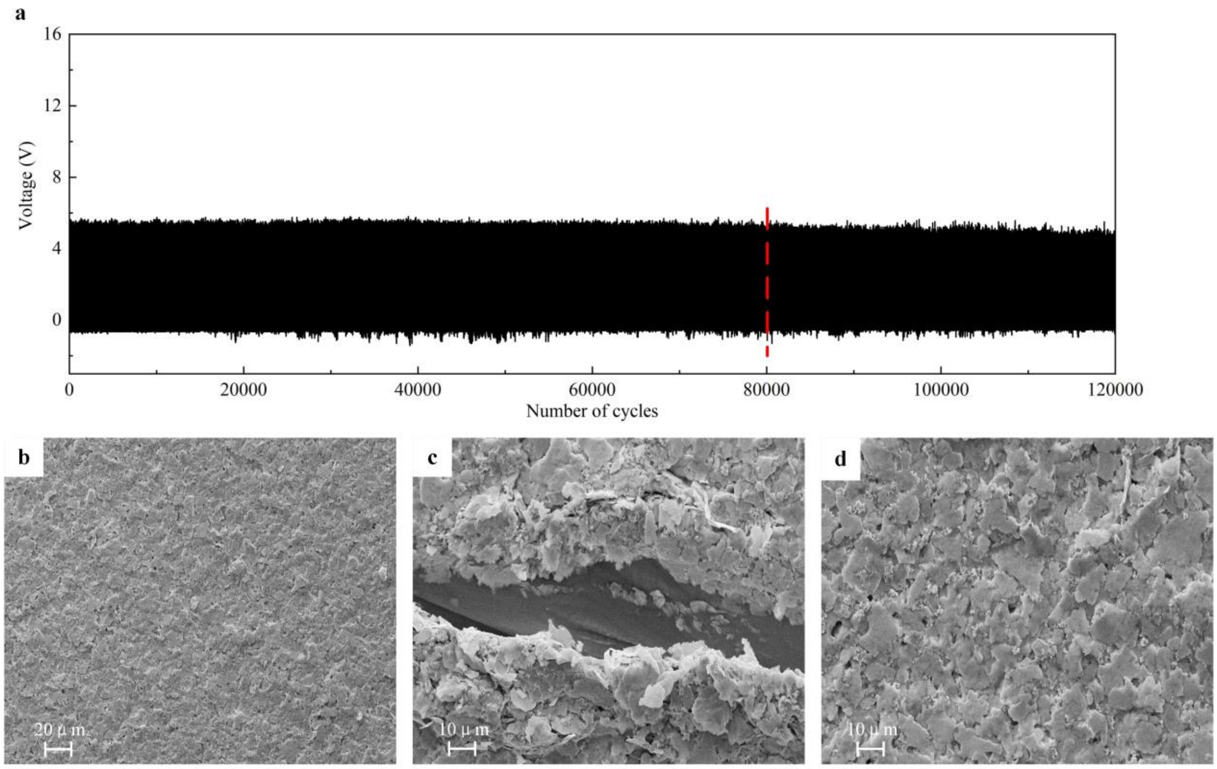

Figure S4. The SEM images of the electrode surface before and after long-term durability test. a) The durability test after 120,000 cycles. The voltage output of the top sensory PVDF begins to decrease after 80,000 cycles under a normal force of an amplitude of 15 N and frequency of 30 Hz. b) The surface of the silver electrode before the test. c) The silver electrode surface of the top sensory layer after the test of 120,000 cycles. d) The silver electrode surface of the bottom sensory layer after the test of 120,000 cycles.

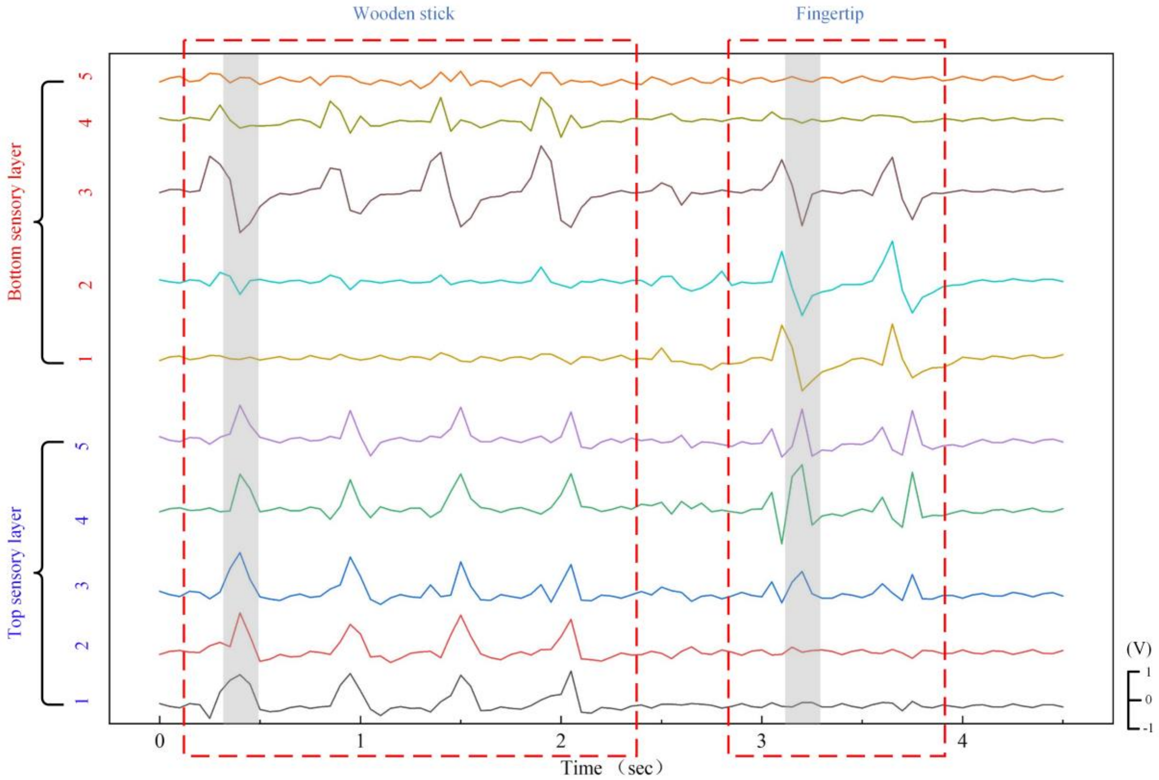

Figure S5. The real-time output voltage waveform of the top sensory layer and bottom sensory layers under multi-touch stimuli mode. The left is the waveform stimulate by a wooden stick and the right by a fingertip. We can obtain the touch positions by reading the signal of the top sensory layer and bottom sensory layer, respectively. For example, at the time of 0.4 seconds (shadow area), the touch stimuli occur at (1, 3), (2, 3), (3, 3), (4, 3), (5, 3) (The first coordinate represents the top sensory layer and the second corresponds to the bottom sensory).

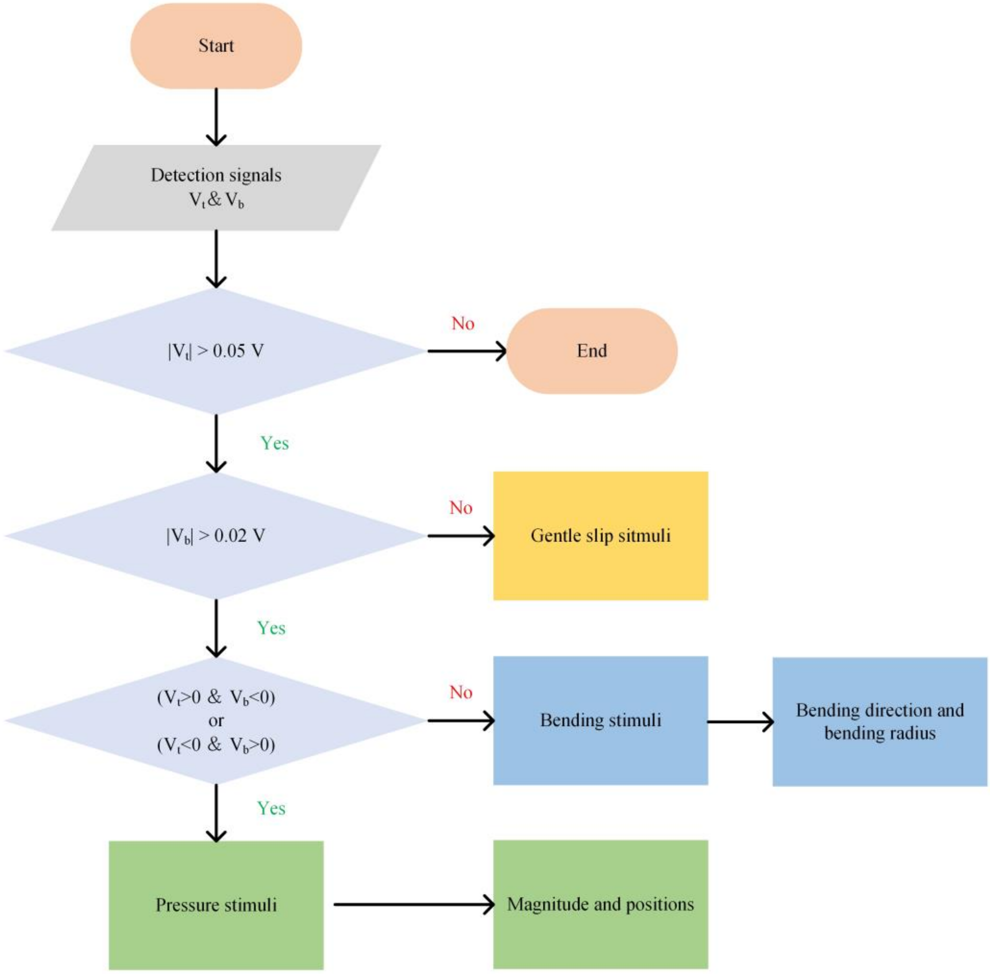

Figure S6. Logical flow diagram to identity diverse external stimuli and calculate the corresponding magnitude, positions or bending direction and radius.

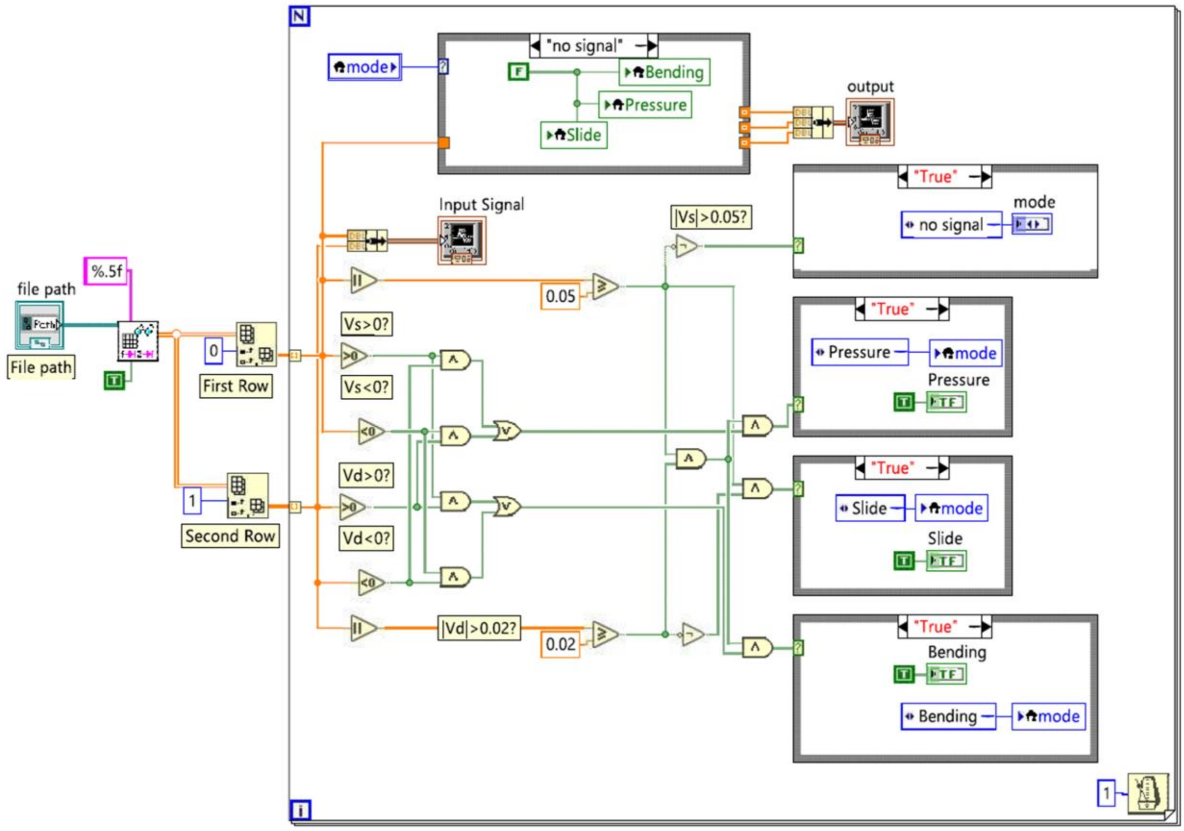

Figure S7. Diagram of the logical circuit developed in LabVIEW 2017. All pixels are scanned by the logical circuit. For each pixel, the logical circuit simultaneously decides the locations and modes of the external stimuli and displays the voltage signals representing pressure magnitude or bending direction and radius.

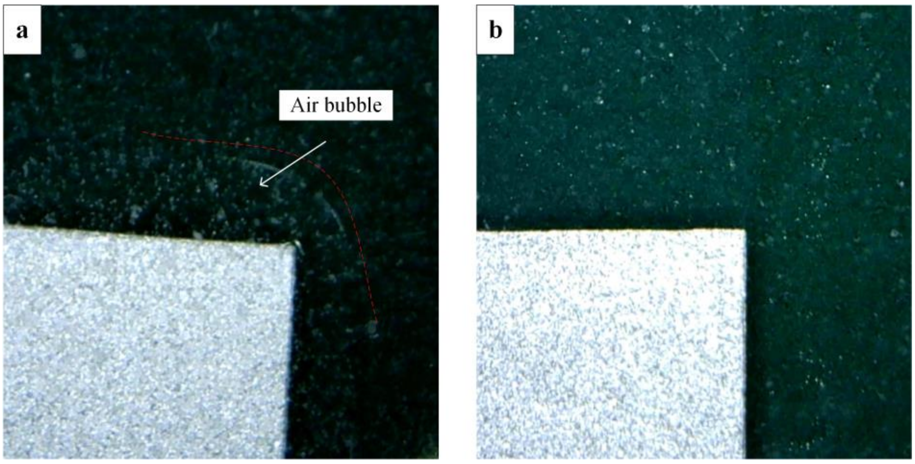

Figure S8. The influence of different packaging methods on the adhesion between each layer of the tactile sensor array. a) The air bubble occurs at the interface between the PVDF layer and the PDMS layer when the PDMS film covers the PVDF film directly. b) Spin-Coating PDMS solution eliminates the bubbles.

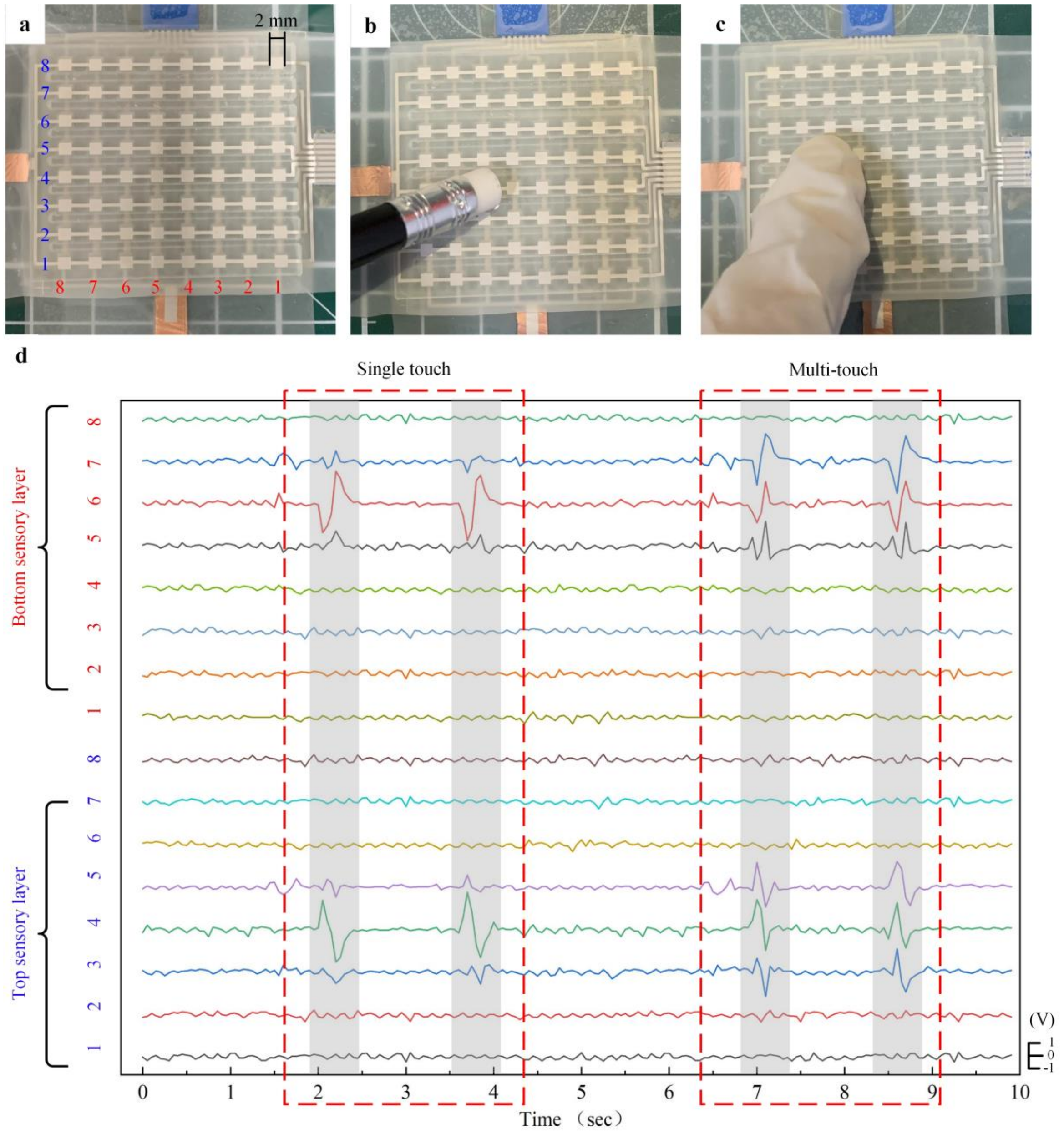

Figure S9. Illustration of the scalability of the design. a) Photograph of the fabricated 8×8 tactile sensor array with each unit of 2×2 mm^2. b) Detection of a signal touchpoint. c) Detection of multiple touchpoints. d) The real-time output voltage waveforms of the top sensory layer and bottom sensory layers corresponding to a signal touchpoint and multiple touchpoints.

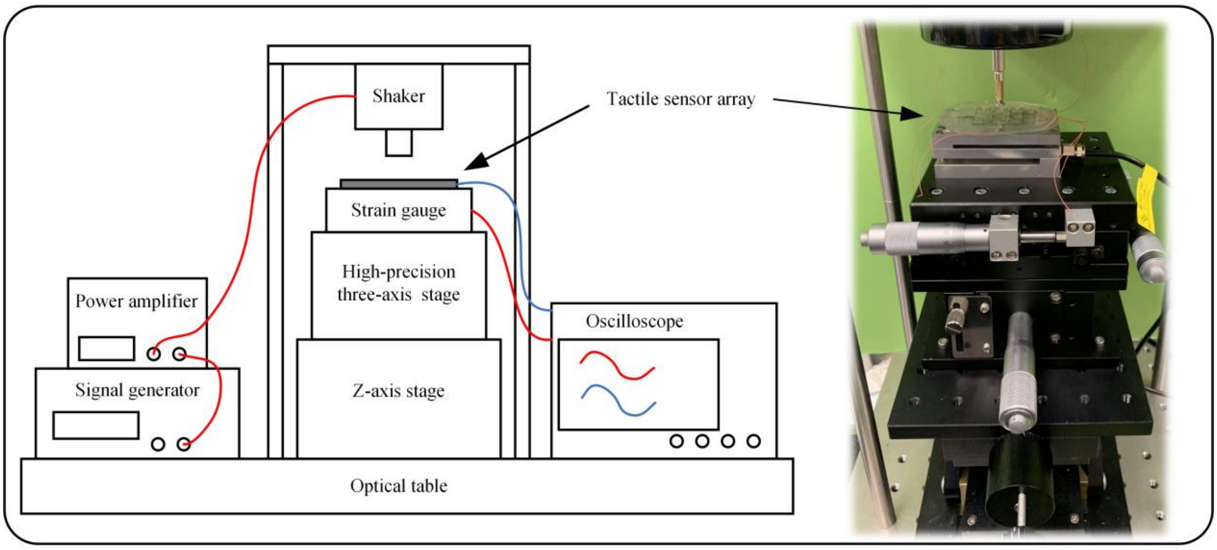

Figure S10. The experimental setup for the pressure sensing tests. The sensor array is mounted to a high-precision three-axis stabilized platform. To ensure the horizontality of the platform, we fix the platform onto an optical table. A shaker (Gelsonlab, HSPW-003) driven by a signal generator (RIGOL Technologies, DG1062Z) and power amplifier (Shenzhen TZT Technology co., Ltd, FPA101A) provides the pressure. The pressure is measured by a high-resolution strain gauge (SIMBATOUCH, SBT291) which is attached to the bottom of the sensor array. The electrical signal generated by the sensor array is recorded by an oscilloscope (Rohde&Schwarz, RTE 1024).

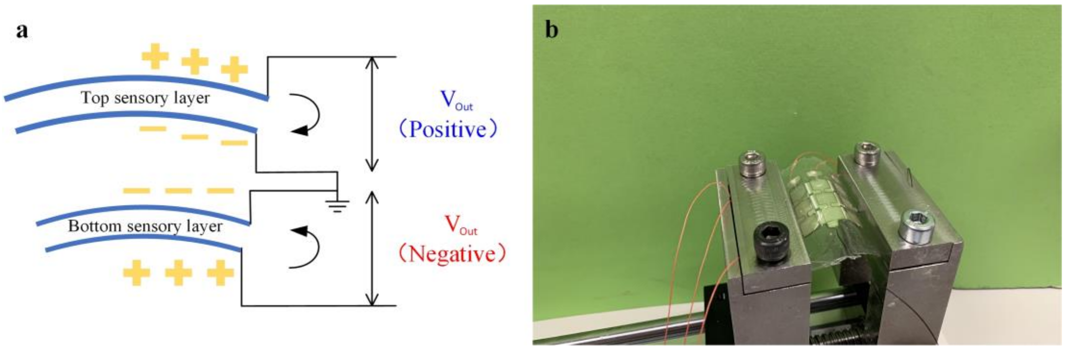

Figure S11. The experimental setup for the bending sensing tests. a) Circuit connection in bending tests. b) The tactile sensor array is fixed onto two fixtures.

Table S1. Comparison of the existing tactile sensors with our work. It can be found that the proposed tactile system has the superiority of real-time detection and differentiation of diverse external stimuli.

**Tactile sensor type**		**Response time**	**Diverse stimuli**			**Identification**	**Electrode Structure**
			**Normal Force**	**Bending Radius**	**Bending Direction**		
Capacitive	Pyramidal microstructure [1]	<40ms	Yes	-[e]	-	-	Row+column [a]
	Mutual capacitive coupling [2]	Not Given	Yes	Yes	-	Historical waveform	Row+column
	Dual-layer capacitor [3]	>200ms	Yes	Yes	-	Real time for slow responses	Row+column
Piezoresistive	Hollow-sphere microstructure [4]	<47ms	Yes	-	-	-	Row+column
	Interlocked zinc oxide nanorod [5]	<14ms	Yes	Yes	-	Historical waveform	Two-electrode[b]
	Interlocked Microdome [6]	<18ms	Yes	Yes	-	Historical waveform	Row+column
	Dual-layer CNT-PU [7]	Not given	Yes	Yes	Yes	Real time	/
Piezoelectric	Field-effect transistor [8]	Not given	Yes	-	-	-	Two-electrode[c]
	Flat film [9]	<55ms	Yes	-	-	-	Single-electrode
	Interlocked microridge [10]	<10ms	Yes	Yes	-	Historical waveform	Two-electrode
	Dual-layer piezoelectric films [11]	Not given	Yes	Yes	Yes	Historical waveform	/[d]
Triboelectric	Multilayered thermoplastic polyurethane [12]	<1.4 ms	Yes	-	-	-	Single-electrode
	Pyramidal microstructure [13]	<514 ms	Yes	Yes	-	Historical waveform	-
	Microstructure [14]	<100 ms	Yes	Yes	-	Historical waveform	Single-electrode
**Human skin**	-	**<40ms**	**Yes**	**Yes**	**Yes**	**Real time**	**/**
**Our work**	**Dual-layer PVDF films**	**<10ms**	**Yes**	**Yes**	**Yes**	**Real time**	**Row+column**

a) Row+column electrode structures use one wire for each column or row, thus decreasing the number of the wires used in the devices and reducing the burden of the wire arrangements; b) For single electrode structures, the sensing units share the same wire for one side and they respectively connect to independent wires for the other side; c) For two-electrode structures, each sensing unit respectively connects to two independent wires; d) The symbol '/' represents the structures only contain one sensing unit; e) The symbol '-' represents "Not Applicable".

Table S2. The measured output peak voltage of the top PVDF sensory layer with different bending direction α and bending radius R.

R \ α	0°	30°	45°	60°	90°
10 mm	0.9320 V	0.5826 V	0.4437 V	0.3365 V	0.1740 V
15 mm	0.7262 V	0.4798 V	0.3323 V	0.2796 V	0.1467 V
20 mm	0.5270 V	0.3684 V	0.2561 V	0.2141 V	0.1183 V
25 mm	0.3768 V	0.2485 V	0.1836 V	0.1401 V	0.0858 V
30 mm	0.2447 V	0.1201 V	0.1182 V	0.0575 V	0.0504 V

Table S3. The measured output peak voltage of the bottom PVDF sensory layer with different bending direction α and bending radius R.

R \ α	0°	30°	45°	60°	90°
10 mm	0.1619 V	0.3081 V	0.3991 V	0.5541 V	0.8895 V
15 mm	0.1103 V	0.2539 V	0.3051 V	0.4531 V	0.7349 V
20 mm	0.0793 V	0.1940 V	0.2308 V	0.3463 V	0.5431 V
25 mm	0.0632 V	0.0128 V	0.1744 V	0.2338 V	0.3563 V
30 mm	0.0433 V	0.0572 V	0.1105 V	0.1157 V	0.2198 V

Table S4. Fitting equations and the corresponding coefficients in Figure 4a,b. *V* is the output voltage, *R* is bending radius, *α* is bending direction. The *r*-square indicates the strong correlation between the fitting results and experimental results.

$V=b_0+b_1\times R+b_2\times\alpha+b_3\times R^2+b_4\times\alpha^2+b_5\times\alpha\times R$							
	b_0	b_1	b_2	b_3	b_4	b_5	*r*-square
Top sensory layer	1.2503	-0.0364	-0.0142	8.7041E-5	3.2727E-5	3.1378E-4	0.9936
Bottom sensory layer	0.1842	-0.0061	0.0084	6.7189E-5	3.3824E-5	-3.1793E-4	0.9925

References

[1] C. M. Boutry, A. Nguyen, Q. O. Lawal, A. Chortos, S. Rondeau-Gagné, Z. Bao, *Adv. Mater.* **2015**, *27*, 6954.

[2] M. S. Sarwar, Y. Dobashi, C. Preston, J. K. M. Wyss, S. Mirabbasi, J. David, W. Madden, *Sci. Adv.* **2017**, *3*, 1.

[3] H. Jin, S. Jung, J. Kim, S. Heo, J. Lim, W. Park, H. Y. Chu, F. Bien, K. Park, *Sci. Rep.* **2017**, *7*, 1.

[4] L. Pan, A. Chortos, G. Yu, Y. Wang, S. Isaacson, R. Allen, Y. Shi, R. Dauskardt, Z. Bao, *Nat. Commun.* **2014**, *5*, DOI 10.1038/ncomms4002.

[5] M. S. Suen, Y. C. Lin, R. Chen, *Sensors Actuators, A Phys.* **2018**, *269*, 574.

[6] J. Park, Y. Lee, J. Hong, Y. Lee, M. Ha, Y. Jung, H. Lim, S. Y. Kim, H. Ko, *ACS Nano* **2014**, *8*, 12020.

[7] H. Chen, Z. Su, Y. Song, X. Cheng, X. Chen, B. Meng, Z. Song, D. Chen, H. Zhang, *Adv. Funct. Mater.* **2017**, *27*, DOI 10.1002/adfm.201604434.

[8] N. T. Tien, S. Jeon, D. Il Kim, T. Q. Trung, M. Jang, B. U. Hwang, K. E. Byun, J. Bae, E. Lee, J. B. H. Tok, Z. Bao, N. E. Lee, J. J. Park, *Adv. Mater.* **2014**, *26*, 796.

[9] C. Deng, W. Tang, L. Liu, B. Chen, M. Li, Z. L. Wang, *Adv. Funct. Mater.* **2018**, *28*, 1.

[10] M. Ha, S. Lim, S. Cho, Y. Lee, S. Na, C. Baig, H. Ko, *ACS Nano* **2018**, *12*, 3964.

[11] H. J. Lee, S. Y. Chung, Y. S. Kim, T. Il Lee, *Nano Energy* **2017**, *38*, 232.

[12] K. Zhou, Y. Zhao, X. Sun, Z. Yuan, G. Zheng, K. Dai, L. Mi, C. Pan, C. Liu, C. Shen, *Nano Energy* **2020**, *70*, 104546.

[13] G. Zhao, Y. Zhang, N. Shi, Z. Liu, X. Zhang, M. Wu, C. Pan, H. Liu, L. Li, Z. L. Wang, *Nano Energy* **2019**, *59*, 302.

[14] Y. Yang, H. Zhang, Z. H. Lin, Y. S. Zhou, Q. Jing, Y. Su, J. Yang, J. Chen, C. Hu, Z. L. Wang, *ACS Nano* **2013**, *7*, 9213.
